# A Single Surgeon's Experience with Open, Laparoscopic, and Robotic Partial Nephrectomy

**DOI:** 10.1155/2014/430914

**Published:** 2014-10-29

**Authors:** Zachary Klaassen, Robert M. Kohut, Dhruti Patel, Martha K. Terris, Rabii Madi

**Affiliations:** ^1^Department of Surgery, Section of Urology, Medical College of Georgia, Georgia Regents University, 1120 15th Street, BA 8414, Augusta, GA 30912, USA; ^2^The Urology Institute, University Hospitals Case Medical Center, Case Western Reserve University, Cleveland, OH 44106, USA

## Abstract

*Objective*. To report the perioperative outcomes of patients treated with partial nephrectomy by a single surgeon using three surgical modalities—open, laparoscopic, and robotic. *Methods*. Between August 2006 and February 2012, 106 consecutive patients underwent open partial nephrectomy (OPN) (*n* = 23), laparoscopic partial nephrectomy (LPN) (*n* = 48), and robotic partial nephrectomy (RPN) (*n* = 35) by a single surgeon. Clinical variables, operative parameters, and renal functional outcomes were analyzed. *Results*. Preoperative patient characteristics were similar except for baseline glomerular filtration rate (GFR), which was highest in the RPN group (*P* = 0.004). Surgery time was longest in the RPN group (244 minutes) and shortest in the OPN group (163 minutes, *P* < 0.0001). Patients who had OPN had the highest incidence of 30-day complications (30%), while the RPN approach had the lowest (14%, *P* = 0.008). *Conclusions*. When performed by a single surgeon, robotic partial nephrectomy appears to be associated with fewer complications than both open and laparoscopic partial nephrectomy. Kidney function was not affected by surgical approach.

## 1. Introduction

In 2014, there will be 63,920 new cases of kidney and renal pelvis cancer diagnoses resulting in approximately 13,860 deaths [1]. Renal cell carcinoma (RCC) is one of the most lethal genitourinary cancers and comprises 85% of these new diagnoses [2]. The incidence of RCC has steadily increased over the past few decades due to incidentally discovered small renal masses (SRM) on radiographic imaging resulting in stage migration to clinical T1a disease from more advanced disease [3]. Numerous treatment options exist for clinical T1a disease, including total nephrectomy, partial nephrectomy, radiofrequency ablation, cryoablation, and high-intensity focused ultrasound (HIFU).

In 2009, the American Urologic Association (AUA) published the “Guideline for the Clinical Stage 1 Renal Mass” which concluded that partial nephrectomy is the standard of care for clinical T1a disease suggesting that the open approach is preferred over minimally invasive techniques [4]. In experienced hands, laparoscopic partial nephrectomy (LPN) is associated with similar oncologic outcomes to open partial nephrectomy (OPN) with the added benefit of decreased narcotic use, decreased hospital stay, and earlier convalescence [5–7]. Robotic partial nephrectomy (RPN) and LPN have also been compared with similar outcomes [8], if not superior RPN outcomes [9], in the hands of an experienced minimally invasive surgeon.

Most studies report the outcomes of either (a) single surgeon and single and/or dual approach or (b) multicenter, multisurgeon, and dual approach. One variable that is typically not accounted for is the surgeon. Surgeon's variability could be a major confounder in those studies. To our knowledge, there have been no reports of a single surgeon's experience with each of the three modalities. Our hypothesis is that when performed by a single surgeon, open, laparoscopic, and robotic partial nephrectomy could differ in postoperative complication rates and overall kidney function. Thus, we report the perioperative outcomes of 106 consecutive patients treated with partial nephrectomy by a single surgeon using all three surgical approaches—OPN, LPN, and RPN.

## 2. Materials and Methods

### 2.1. Patients

After obtaining Institutional Review Board approval, a retrospective chart review of a prospectively collected kidney cancer database was performed. From August 2006 to February 2012, a single surgeon (RM) with minimally invasive and urologic oncology fellowship training performed 106 consecutive partial nephrectomies using any of the three available surgical modalities—OPN, LPN, or RPN. The decision for the approach was multifactorial and was based on patient and tumor characteristics. Initially in the practice, LPN was offered to patients with tumors of favorable size and location. OPN was reserved for patients who were uninephric, had compromised kidney function, or had large tumors in an unfavorable location. In early 2008, RPN gradually replaced LPN as the preferred minimally invasive approach. Preoperative variables and demographics analyzed included age, gender, body mass index (BMI), American Society of Anesthesiologist (ASA) score, glomerular filtration rate (GFR), hematocrit (within 30 days of surgery), tumor laterality, and tumor size. Renal nephrometry score was calculated as previously described [10]. Operative variables analyzed included operative time, estimated blood loss (EBL), complications, pathology (benign, malignant), and margin status. Postoperative variables included length of stay, discharge hematocrit, transfusion, GFR, 3-month GFR, and 30-day complications. The indication for partial nephrectomy was an enhancing renal mass found on cross-sectional abdominal imaging. Patients were followed postoperatively for evidence of immediate and delayed adverse events. Pathology was reviewed by a dedicated genitourinary pathologist for margin status and tumor type.

### 2.2. Surgical Technique

*OPN*. Briefly, patients were positioned in a modified 45-degree flank and an incision was made for access to the retroperitoneum. The kidney was dissected free of attachments in the retroperitoneal space and the hilum was identified. Intravenous mannitol (12.5 g) administration and renal hypothermia (cold ischemia) were utilized prior to hilar clamping in all cases. The hilum was clamped using the Satinsky vascular clamp and the resection of the renal lesion was performed with sharp dissection. The collecting system was closed primarily, and individual vessels were identified and controlled using nonabsorbable monofilament suture. Argon beam coagulation achieved hemostasis prior to reapproximation of the renal parenchyma with Surgicel (Ethicon Inc, Somerville, NJ) bolsters. In all cases, a percutaneous drain was placed.

*LPN*. Patients in this series have undergone transperitoneal or retroperitoneal LPN. The patient was positioned in a modified flank configuration with a total of four ports. Once the hilum and mass were identified, mannitol (12.5 g) was administered and the hilum was controlled using a laparoscopic vascular clamp (warm ischemia). Subsequently, the tumor was excised using endoscopic shears. Surgicel (Ethicon Inc, Somerville, NJ) and FloSeal (BioSurgery, Deerfield, IL) were used for hemostasis and the tumor bed was repaired in a running fashion based on the depth of the lesion. The parenchyma was reapproximated with 2.0 v lock suture, Surgicel (Ethicon Inc, Somerville, NJ) bolsters, and secured with Hem-o-lok clips. In superficial tumors penetrating less than 1 cm into kidney parenchyma, clamping and/or repair of the base of resection was not performed.

*RPN*. Robotic-assisted LPN was performed using the da Vinci robotic surgical system. Patients were placed in a standard 60–70-degree modified flank position. All approaches were transperitoneal. All patients underwent warm ischemia, which was achieved using either laparoscopic bulldog clamps or a vascular clamp. Likewise, mannitol (12.5 g) was given prior to hilar clamping. Similar to the LPN technique, the resection bed was closed in a running fashion using an absorbable suture and the defect was approximated using a Surgicel (Ethicon Inc, Somerville, NJ) bolster in most cases.

### 2.3. Statistical Analysis

The Statistical Package for the Social Sciences v.19 (SPSS Inc., Chicago, IL) was used to perform all statistical analyses. All *P* values were one-sided, and *P* < 0.05 was considered statistically significant. Categorical variables were compared using a Chi-square test and continuous variables were compared using one-way ANOVA.

## 3. Results

Among the 106 patients in the study, 23 patients (22%) underwent OPN, 48 patients (45%) underwent LPN, and 35 patients (33%) underwent RPN.

### 3.1. Preoperative Variables and Demographics

There was no difference in age, gender, BMI, ASA score tumor laterality, or tumor size between the three groups (Table 1). There was a statistical trend towards lower preoperative hematocrit for OPN patients compared to the minimally invasive patients (OPN—40 ± 4%, LPN—43 ± 4%, RPN—42 ± 4%, *P* = 0.09). Preoperative GFR was 71 ± 28 mL/min for OPN patients, 76 ± 25 mL/min for LPN patients, and 96 ± 37 mL/min for RPN patients (*P* = 0.004). Patients undergoing RPN had lower mean nephrometry scores (*P* = 0.01).

### 3.2. Operative Outcomes

There was no difference in intraoperative complication rate, tumor pathology, or margin status between the OPN, LDN, and RPN patients (Table 2). Operative time was the shortest in the OPN group (OPN—163 ± 35 min, LPN—227 ± 53 min, RPN—244 ± 53 min, *P* < 0.0001). EBL (OPN—367 ± 286 mL, LPN—163 ± 179 mL, RPN—191 ± 173 mL, *P* < 0.0001) was significantly different between the three groups of patients. All patients who had OPN had the kidney cooled for an average time of 8 minutes before excision of the tumor. There were no conversions from LPN or RPN to OPN. Mean console time for RPN was 207 minutes.

### 3.3. Postoperative Outcomes

There were no differences in length of stay, blood transfusion, discharge GFR, or 3-month GFR for the three cohorts of patients (Table 3). Discharge hematocrit was significantly lower for OPN patients (30 ± 3%) compared to LPN (36 ± 4%) and RPN (35 ± 4%) patients (*P* < 0.0001). Upon follow-up, the OPN group had the highest incidence of 30-day complications (30%), while the RPN group had the lowest (14%, *P* = 0.008). The OPN complications included Clavien I (*n* = 2), Clavien II (*n* = 1), Clavien III (*n* = 3), and Clavien IV (*n* = 1). The LPN complications included Clavien I (*n* = 4), Clavien II (*n* = 3), and Clavien III (*n* = 1). The RPN complications included Clavien I (*n* = 1), Clavien II (*n* = 1), and Clavien III (*n* = 3). Specific 30-day complications for each group are listed in Table 4.

## 4. Discussion

Radical nephrectomy (RN) has been the mainstay of treatment for clinical stage I tumors resulting in excellent cancer specific survival, local tumor control, and progression free survival; however, reports have highlighted a negative impact on renal function and chronic kidney disease (CKD) associated with RN [11]. This effect was acknowledged in the 2009 AUA guideline for the management of small renal masses which concluded that OPN should be considered standard of care and that minimally invasive options, such as LPN and RPN, be considered as second line treatment modalities [4]. Furthermore, recent studies have established that partial nephrectomy (PN) provides similar oncological outcomes when compared to RN [12–14].

The current study is the first, to our knowledge, which compares all three modalities of PN (OPN, LPN, and RPN) by a single surgeon. Multi-institutional studies may represent dissimilar patient cohorts that introduce selection bias and may invoke concerns regarding surgeon variability [8]. A single surgeon evaluation allows minimally invasive modalities to be compared with a “control” group of OPN patients operated on by the same surgeon. In the current study, there was no randomization or objective criteria for the PN approach; rather, the trend was for more minimally invasive surgery as the surgeon developed a comfort level with the LPN and RPN approach. This is evident by the fact that tumor characteristics for each group are comparable (laterality, size, pathology, and margin status).

Previous single surgeon studies have analyzed RPN versus LPN [11, 15–19] with the consensus being that RPN offers a shorter learning curve to minimally invasive PN and comparable warm ischemia time, EBL, and length of stay. Two common criticisms associated with RPN include (i) the cost of the robot and (ii) increased surgical time. Although it is difficult to mitigate the cost associated with RPN compared to LPN and OPN, the oft-held conception that procedures take significantly longer may be a misconception after achieving the learning curve associated with RPN. Lavery et al. concluded that RPN achieved similar operative times to LPN after only 5 procedures and the current study corroborates similar operative times between the two approaches (244 ± 53 versus 227 ± 53 min) [19].

In the OPN cohort, there was a 30-day complication rate of 30%, which was statistically significant compared to the LPN (17%) and RPN (14%) groups, and higher than that reported by other authors. Gill et al. reported a 16% and 13% postoperative complication rate in their initial comparison of LPN and OPN, respectively [20]. Bhayani and Waxman and Winfield reported postoperative complication rates of 16% and 18.2%, respectively, in each of their single-surgeon experiences with LPN [21, 22]. A recent report, which pooled data from 4 institutions considered centers-of-excellence, reported a postoperative complication rate of 14.4% in patients who underwent RPN. Most complications were Clavien grade I/II and were managed conservatively and affirm the safety of RPN in experienced hands [23]. The complication rate was lowest in the RPN group at 14% and is similar to other reports, with ranges of 10–20% [8, 15, 24].

The primary limitation of the study is the inherent selection bias and patient confounders associated with a retrospective study. Surgical confounders were minimized as only operations performed by a single surgeon were analyzed. Secondly, renorrhaphy differed between the OPN and minimally invasive cohorts along with the use of renal cooling in the OPN group compared to warm ischemia time for the minimally invasive approaches. Furthermore, we acknowledge the small sample sizes amongst the three groups.

## 5. Conclusions

Nephron sparing surgery has emerged as the optimal surgical approach for T1a and in experienced hands T1b kidney masses. By removing surgeon as a confounding variable, comparison between various surgical techniques becomes more meaningful. Through technological advancement, OPN has given way to LPN and RPN. Although not currently the gold standard for partial nephrectomy, robotic surgery continues to emerge as the future for renal surgery secondary. RPN offers less blood loss and fewer postoperative complications than OPN and overcomes the technical difficulties of LPN. It is currently our standard approach for nephron sparing surgery.

## Figures and Tables

**Table 1:** Preoperative demographics.

Preoperative variable	OPN (*n* = 23)	LPN (*n* = 48)	RPN (*n* = 35)	*P* value^*^
Mean age ± SD (years)	62 ± 10	56 ± 14	56 ± 14	0.20
Male/female	15/8	30/18	21/14	0.88
Mean BMI ± SD (kg/m^2^)	28.9 ± 7.9	31.9 ± 7.6	32.8 ± 14.7	0.37
Mean ASA score ± SD	2.4 ± 0.5	2.5 ± 0.5	2.5 ± 0.5	0.91
Mean GFR ± SD (mL/min)	71 ± 28	76 ± 25	96 ± 37	0.004
Mean hematocrit ± SD (%)	40 ± 4	43 ± 4	42 ± 4	0.09
Tumor laterality, left/right	10/13	24/24	15/20	0.81
Mean tumor size ± SD (cm)	3.8 ± 1.1	3.0 ± 1.9	3.6 ± 2.0	0.13
Mean nephrometry score ± SD	9.0 ± 2.2	8.7 ± 2.0	7.4 ± 2.1	0.01

OPN: open partial nephrectomy; LPN: laparoscopic partial nephrectomy; RPN: robotic partial nephrectomy; BMI: body mass index; ASA: American Society of Anesthesiologists; GFR: glomerular filtration rate; NS: not significant.

^*^Statistical significance to <0.05.

**Table 2:** Operative variables.

Operative variable	OPN (*n* = 23)	LPN (*n* = 48)	RPN (*n* = 35)	*P* value^*^
Mean operative time ± SD (min)	163 ± 35	227 ± 53	244 ± 53	<0.0001
Mean EBL ± SD (mL)	367 ± 286	163 ± 179	191 ± 173	<0.0001
Intraoperative complications	2	0	4	0.38
Pathology, malignant/benign	21/2	36/12	30/5	0.21
Margin status (positive/negative)	0/23	3/45	2/33	0.44

OPN: open partial nephrectomy; LPN: laparoscopic partial nephrectomy; RPN: robotic partial nephrectomy; EBL: estimated blood loss.

^*^Statistical significance to <0.05.

^1^Cold ischemic time for OPN and warm ischemic time for LPN and RPN.

**Table 3:** Postoperative variables.

Postoperative variable	OPN (*n* = 23)	LPN (*n* = 48)	RPN (*n* = 35)	*P* value^*^
Mean length of stay ± SD (days)	3.0 ± 1.8	1.9 ± 1.7	2.6 ± 5.1	0.38
Mean discharge hematocrit ± SD (%)	30 ± 3	36 ± 4	35 ± 4	<0.0001
Transfusion, yes/no	1/23	1/47	0/35	1.00
Mean discharge GFR ± SD (mL/min)	64 ± 32	67 ± 26	79 ± 30	0.12
Mean 3-month GFR ± SD (mL/min)	61 ± 26	70 ± 30	76 ± 25	0.20
30-day complication, *N* = (%)	7 (30)	8 (17)	5 (14)	0.008

OPN: open partial nephrectomy; LPN: laparoscopic partial nephrectomy; RPN: robotic partial nephrectomy; GFR: glomerular filtration rate.

^*^Statistical significance to <0.05.

**Table 4:** 30-day complication rates.

30-day complications	OPN (*n* = 23)	LPN (*n* = 48)	RPN (*n* = 35)
Delayed bleeding	1	3	3
Urine leak	2	1	0
Cardiovascular	2	0	0
Respiratory	2	3	0
Ileus	0	2	1
Other	0	0	1
